# Efficient isolation of *Magnolia* protoplasts and the application to subcellular localization of *MdeHSF1*

**DOI:** 10.1186/s13007-017-0193-3

**Published:** 2017-05-23

**Authors:** Yamei Shen, Dong Meng, Kim McGrouther, Junhong Zhang, Lailiang Cheng

**Affiliations:** 1 School of Landscape and Architecture, Zhejiang A & F University, Lin’an, 311300 Zhejiang China; 2 Department of Horticulture, Cornell University, Ithaca, NY 14853 USA; 3 Scion, Private Bag 3020, Rotorua, 3046 New Zealand; 4 State Key Laboratory of Subtropical Silviculture, Zhejiang A & F University, Lin’an, 311300 Zhejiang China

**Keywords:** *Magnolia*, Protoplast isolation, Heat shock transcription factor, Heat stress, Subcellular localization

## Abstract

**Background:**

*Magnolia* is a woody ornamental plant, which is widely used in urban landscaping. However, its lengthy juvenile period and recalcitrance to regeneration impedes functional characterization of its genes.

**Results:**

We developed an efficient protoplast isolation and transient expression system for *Magnolia denudata* × *Magnolia acuminata* ‘Yellow River’. The highest yield of protoplasts was obtained from young leaves digested in 3% Cellulase R10, 0.8% Macerozyme R10, 0.04% pectinase and 0.4 M mannitol enzymolysis solution for 6 h. For transfection of protoplasts, 20% PEG4000 for 5 min was optimal. To verify the protoplast system and begin to understand heat tolerance in *Magnolia*, a heat shock transcription factor *MdeHSF1* was cloned from ‘Yellow River’, which belongs to the HSF subfamily A and has significant homology with *AtHSFA1A*. Subcellular localization analysis indicated that *MdeHSF1* was expressed in the cell nucleus. Furthermore, qPCR analysis of the *MdeHSF1* transcript level in response to high temperature stress suggested that *MdeHSF1* might be involved in regulating heat stress tolerance in ‘Yellow River’.

**Conclusion:**

The described protocol provides a simple and straightforward method for isolating protoplast and exploring gene subcellular localization of *MdeHSF1* in *Magnolia*. This expands the new research of protoplast isolation and transfection in *Magnolia*.

**Electronic supplementary material:**

The online version of this article (doi:10.1186/s13007-017-0193-3) contains supplementary material, which is available to authorized users.

## Background

*Magnolia* is among the best known ornamental plant used in landscaping. The majority of *Magnolia* species are distributed in America and East Asia, including relict plants, some of which have survived for hundreds of millions of years [1]. Many species of *Magnolia* growing in the wild are endangered, and some face extinction. Several factors have contributed to this situation, including threats to their habitats, limited genetic diversity, and difficulties associated with breeding. Efforts are being made to protect, develop, and spread magnolias. Grafting and seeding are the main methods for propagating *Magnolia*. However, *Magnolia* has a long juvenile period (4–5 years or more) and is recalcitrant to regeneration. Currently, lack of any transformation system has become a limiting step for functional characterization of its genes including subcellular localization, protein–protein interaction, DNA–protein interaction, and transgenic studies [2–4]. Thus, transformation methods are urgently needed.

The plant protoplast can serve as a versatile cell-based experimental system to analyze gene expression during a transient time period [3, 5, 6]. Macromolecules, such as DNA, RNA and proteins, can be delivered into protoplasts using various methods, e.g., PEG-calcium fusion [7], electroporation [7] and microinjection [8]. Signal transduction and metabolic pathways as well as the transcription and translation machinery can be transiently manipulated to investigate cell-autonomous regulation and responses [9]. In addition, protoplasts can be used as a method for breeding. Plant protoplast isolation was first reported half a century ago using the root tip of tomato seedlings [10]. Subsequently, mesophyll cells [11–13], fruits [14, 15], stem [16], seed [17] and calluses [18] have been used as materials to isolate the protoplast. In recent years, protoplast isolation and transfection was well established for *Arabidopsis* [3, 4, 9, 19], *Populus* spp. [20, 21] and *Bambusa vulgaris* [22], etc. However, no published protocol for protoplast isolation and transfection is available for *Magnolia*.

As temperatures continue to rise globally, plants are increasingly exposed to heat stress. The major heat shock factors regulating heat stress response are heat shock transcription factors (HSFs) [23], which are important regulators for sensing and signaling heat stress [24]. HSFs have been characterized in *Brassica rapa* spp. *pekinensis* [23], *Helianthus annuus* [25], *Arabidopsis* [26], *Zea mays* [27], *Glycine max* [28] and *Pyrus bretschneideri* [24]. As most species of *Magnolia* appear to be particularly sensitive to heat stress, characterizing HSFs in *Magnolia* is of great significance to understanding and eventually improving their tolerance to heat stress.

**Fig. 1:**
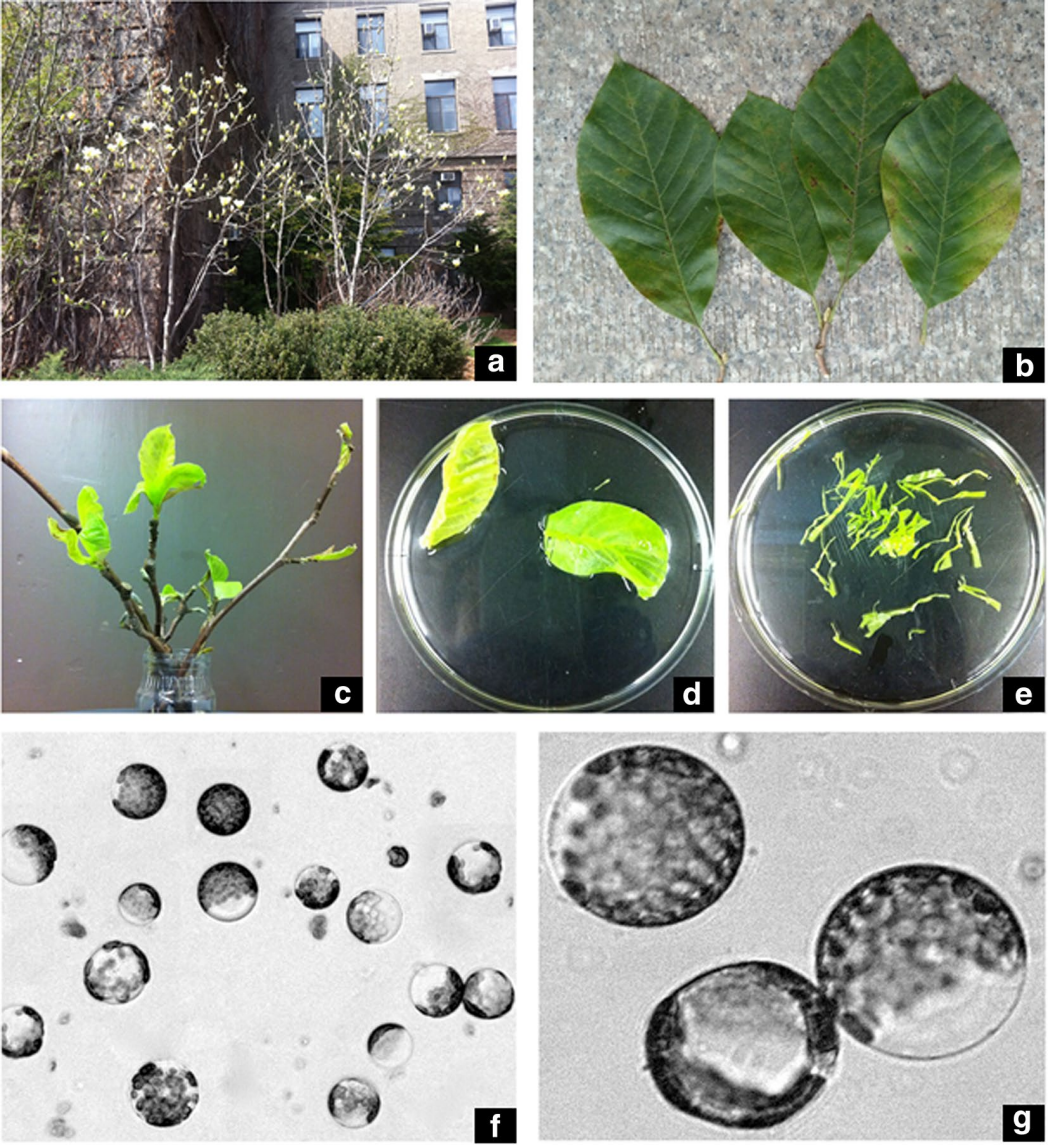
Different tissues for protoplast isolation in ‘Yellow River’. The shape of ‘Yellow River’ (**a**), mature leaves of ‘Yellow River’ (**b**), young leaves of ‘Yellow River’ (**c**), ‘cut-off’ leaves from ‘Yellow River’ (**d**, **e**), Protoplasts isolated from the young leaves (**f**, **g**), *bar* 50 μm

In this study, we selected one classical species of *Magnolia*, *Magnolia denudata* × *M. acuminata* ‘Yellow River’ (‘Yellow River’), as the plant material to develop an efficient protoplast isolation and transfection protocol. ‘Yellow River’ is a hybrid of *M. denudata* × *M. acuminata* (Fig. 1). They have characteristic yellow flowers and are widely used in urban landscaping in China. As ‘Yellow River’ performs better than *M.* × *soulangeana* (Additional file 1: Table S1) in terms of heat stress tolerance, the molecular mechanism responsible for higher heat-stress resistance in ‘Yellow River’ is worth elucidating. The transient transformation system in protoplast is a cell-based functional genomics tool to explore this molecular mechanism. Therefore, we cloned its heat shock transcription factor *MdHSF1* and characterized its subcellular localization using protoplast transfection in this paper, and the results confirm that the transient transformation system is viable. The method reported here for protoplast isolation and transfection of *Magnolia* ‘Yellow River’ lays a good foundation for the breeding of *Magnolia*, and expands the functional gene research in the field of *Magnolia*.

## Methods

### Plant material and growth conditions

*M. denudata* × *M. acuminata* ‘Yellow River’ was grown on the campus of Cornell University in Ithaca, New York, USA (Fig. 1a) was approximately 20 years old, with the diameter at breast height (DBH) of 11.0 cm. Mature leaves were sampled on July 7, 2014 (Fig. 1b). The annual branches with leaf buds were collected on February 16, 2015 (Fig. 1c). The annual branches were taken into the lab, and cultured in water at 23 °C. After one week, the sprouted leaves were used to isolate protoplasts. Meanwhile, the buds were also collected. All these materials were taken from the annual branches and they all faced in one direction. After being detached, mature leaves, young leaves and buds were washed twice with sterile water, and then sterilized with 70% ethanol for 5 s first and then with 0.1% HgCl_2_ for 5 min. Finally, the materials were used to isolate protoplast after being washed 4 times with sterile water (Fig. 1d–g).

### Protoplast isolation

Protoplast isolation was performed following the methods of Wu [3], Zhai [29], Rezazadeh [11] and Guo [20], with some modifications. 2 g of leaves (Mature leaves or young leaves) were sliced to 1–2 mm width in a sterilized petri dish with a fresh razor blade (2 g of buds was also sliced to fragments with a razor blade), then rapidly transferred into 100 mL sterilized beaker which contained 10 mL enzymatic solution (The enzymatic solution was placed at 55 °C for 10 min, cooled to room temperature, and sterilized using a 0.45 µm filter prior to use).

Then three methods were used to treat samples for choosing the optimal way of protoplast isolation: Method 1: Four concentrations mannitol/sorbitol (0.2, 0.4, 0.6 or 0.8 M) were used to treat the samples for getting optimal enzymatic solution (replicated three times each experiment); Method 2: Six combinations with different concentrations of Cellulase R10 and Macerozyme R10 (a) 1% (w/v) Cellulase R10, 0.4% (w/v) Macerozyme R10, 0.04% (w/v) Pectinase; (b) 1.5% (w/v) Cellulase R10, 0.5% (w/v) Macerozyme R10, 0.04% (w/v) Pectinase; (c) 2% (w/v) Cellulase R10, 0.6% (w/v) Macerozyme R10, 0.04% (w/v) Pectinase; (d) 2.5% (w/v) Cellulase R10, 0.7% (w/v) Macerozyme R10, 0.04% (w/v) Pectinase; (e) 3% (w/v) Cellulase R10, 0.8% (w/v) Macerozyme R10, 0.04% (w/v) Pectinase; (f) 3.5% (w/v) Cellulase R10, 0.9% (w/v) Macerozyme R10, and 0.04% (w/v) Pectinase). Each combination was prepared to the appropriate concentration using a solution containing 20 mM KCl; 20 mM MES-KOH (pH 5.7); 10 mM CaCl_2_; 10 mM β-mercaptoethanol and 0.1% (w/v) bovine serum albumin (replicated three times each experiment). The third method was based on the results from the methods 1 and 2, and involved vacuum-infiltrating leaf strips for 30 min and incubated in the enzymatic solution at 25 °C in darkness with rotation (60 rpm) for about 2, 4, 6, 8, 10 or 12 h (the beaker with the leaf strips was covered with parafilm and aluminum foil) (replicated three times each experiment). After digestion, an equal volume of W5 solution (154 mM NaCl, 125 mM CaCl_2_, 5 mM KCl, 2 mM MES-KOH (pH 5.7), sterilized using a 0.45-mm filter) was added. Next, the solution was filtered through a sterilized nylon membrane (200 µm mesh size) and transferred into a 50 mL sterilized centrifuge tube. Then the nylon membrane was washed twice with 10 mL W5 solution and all the filtrate was transferred to a 50 mL centrifuge tube.

Finally, the filtrate was centrifuged at 100*g* for 10 min, and the supernatant was carefully removed using a pipettor. The protoplasts were washed twice with W5 solution and re-suspended to a final concentration of 1 × 10^6^ protoplasts/mL for transfection.

The protoplast yield of different tissue types (mature leaves, young leaves and bud) was also compared. The viability of the isolated protoplasts was also determined with FDA (fluorescein diacetate) dye [30]. The FDA stock solution was added into the isolated protoplasts at a final concentration of 0.01%. After being incubated in darkness for 5 min and washed three times with W5 solution, protoplast viability was checked under a fluorescence microscope.

### Protoplast transfection

The isolated protoplasts were placed on ice, and an equal volume of PEG solution (20% w/v polyethylene glycol, PEG4000, 0.3 M mannitol and 100 mM CaCl_2_) (with 10% PEG4000 as a control, replicated three times) was added. In order to select the optimal concentration of PEG4000, concentrations of 10, 15, 20, 25 and 30% PEG4000, w/v were tested. Next, 20 µg of the pEZS-NL plasmid, which contained a 35S promoter and the coding sequence fused to EGFP and OCS 3′ terminator which can used for transient expression [31] (Isolated by PureLink^®^ Quick Plasmid Miniprep Kit, Invitrogen, cat. no. K210010), 100 µL of isolated protoplasts and 120 µL of the PEG solution were combined and mixed gently in a 2 mL microfuge tube. The mixture was incubated at room temperature for 5, 10, 15 and 30 min in triplicate (to determine the optimum transfection time) before 220 µL of W5 solution was added to terminate the reaction. The mixture was centrifuged at 120*g* for 5 min, and the supernatant was carefully removed with a pipettor. The protoplasts were re-suspended overnight at room temperature in 2 mL of incubation buffer (0.4 mM mannitol, 4 mM MES-KOH (pH 5.7), 20 mM KCl). Finally, the protoplasts were centrifuged at 120*g* for 2 min and re-suspended with an appropriate amount of WI solution (0.5 M mannitol, 4 mM MES-KOH (pH 5.7), 20 mM KCl). A Zeiss LSM 710 fluorescence microscope was used to visualize the EGFP signal. The transformation efficiency was determined by (%) = (fluorescent protoplast number in view/total protoplast number in view) × 100%.

### Note

All data were analyzed by SPSS.19. The data of transfection rate was analyzed through the square root of the arcsine difference.

### RNA isolation and cloning of *MdeHSF1* and construction of its expression vector

RNA was extracted from leaves of ‘Yellow River’ according to a modified CTAB method and digested with DNase I. RNA was detected by electrophoresis. Total RNA was used to synthesize first-strand cDNA using the SuperScript reverse transcriptase with Oligo-dT primer. The cDNA was then used as templates for PCR amplification.

Our preliminary data obtained on ‘Yellow River’ at Hangzhou, Zhejiang Province of China (Additional file 1: Table S1) showed that ‘Yellow River’ has stronger tolerance to heat stress than *M.* × *soulangana*. We also determined the transcript profile of ‘Yellow River’ leaves using RNA-seq in response to heat treatment. Based on the transcriptome data of *M. sinostellata*, the primers were designed, F: 5′-ATGGACGGTGCTCATGGCAGCA-3′, R: 5′-CAGCCTTTGTTTTCTGATGTAAGAA-3′, for homology-based cloning of *MdeHSF1* via PCR. The PCR products were cloned into the pMD19-T simple vector using T4 DNA Ligase, Then the vector was transferred into *E. coli*. DH5a competent cells and grown on the LB medium with Ampicillin. Positive bacterial colonies were detected by PCR using the M13 primer shown in the pMD19-T vector user manual; then sequenced at Cornell’s Biotechnology Resources Center. Finally, *MdeHSF1* was analyzed from the website of http://www.ncbi.nlm.nih.gov/Structure/cdd/wrpsb.cgi, the protein structure was analyzed using the smart website http://smart.embl-heidelberg.de/, the protein prediction was from the website https://www.predictprotein.org/, and *MdeHSF1* was compared with *HSF* of *Arabidopsis thaliana*.

The correct clone of *MdeHSF1* was cloned into the transient expression vector pEZS-NL through double enzyme digestion (*BamHI* and *XhoI*), then the vector was transferred into the DH5a competent cells. The correct clone was detected by PCR using the 35S promoter primer F and HSF primer R. The clone was cultured on LB liquid medium (Ampicillin) at 37 °C with rotation (120 rpm) for 12 h. The plasmid was isolated using PureLink^®^ Quick Plasmid Miniprep Kit.

### RNA extraction and quantitative real-time PCR (qRT-PCR)

‘Yellow River’ and *M.* × *soulangeana* leaf samples were finely grounded in liquid nitrogen, and RNA was extracted using the CTAB method as described by Gasic et al. [32]. After treatment with RQ1 DNase, RNA was quantified using a NanoDrop spectrophotometer, and the RNA integrity was confirmed by agarose gel electrophoresis. One milligram of total RNA was reverse-transcribed into cDNA using the iScript cDNA Synthesis kit. Actin gene served as the internal control. Every qRT-PCR was performed in 3 replicates on an Icycler iQ5 (BioRad) using the SYBR Green Supermix kit (Bio-Rad) according to the instruction manual. Data were analyzed using iQ5 2.0 software (Bio-Rad) with the ddCT method.

### Note

The sources of all relative reagents were showed in Additional file 1: Table S2.

## Results

### An efficient method of protoplast isolation in ‘Yellow River’

**Fig. 2:**
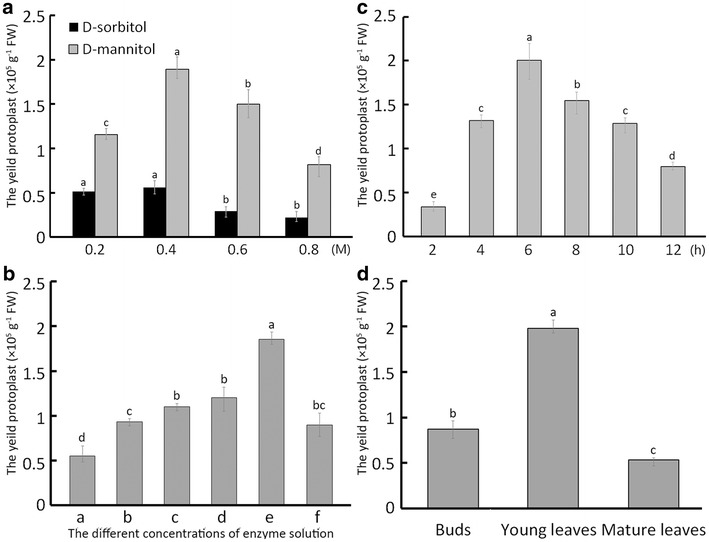
The protoplast yield under different treatments. In response to a range (0.2, 0.4, 0.6 and 0.8 M) of mannitol and sorbitol concentrations in digestion solution with 3% Cellulase R10, 0.8% Macerozyme R10, and 0.4% Pectinase (**a**), protoplast yield as affected by different concentrations of digestion enzymes (**b**): (*a*) 1% (w/v) Cellulase R10, 0.4% (w/v) Macerozyme R10, 0.04% (w/v) Pectinase; (*b*) 1.5% (w/v) Cellulase R10, 0.5% (w/v) Macerozyme R10, 0.04% (w/v) Pectinase; (*c*) 2% (w/v) Cellulase R10, 0.6% (w/v) Macerozyme R10, 0.04% (w/v) Pectinase; (*d*) 2.5% (w/v) Cellulase R10, 0.7% (w/v) Macerozyme R10, 0.04% (w/v) Pectinase; (*e*) 3% (w/v) Cellulase R10, 0.8% (w/v) Macerozyme R10, 0.04% (w/v) Pectinase; (*f*) 3.5% (w/v) Cellulase R10, 0.9% (w/v) Macerozyme R10, and 0.04% (w/v) Pectinase, protoplast yield as affected by digestion time (2, 4, 6, 8, 10 or 12 h) (**c**), protoplast yield as affected by tissue type (buds, young leaves and mature leaves) (**d**)

The yield of protoplast in the treatment of mannitol was significantly higher than that under sorbitol treatment (*p* < 0.01). The optimal concentration was 0.4 M mannitol, which yielded 1.89 × 10^5^ protoplasts/g FW (fresh weight), the highest yield of all the treatments (*p* < 0.01) (Fig. 2a).

Furthermore, we tested the effects of different concentration combinations of cell wall-degrading enzymes on protoplast yield. Via the addition of various concentrations of the enzymes, the protoplast yield tended to increase until the concentration reached 3% (w/v) Cellulase (R10), 0.8% (w/v) Macerozyme (R10) and 0.04% (w/v) Pectinase. The protoplast yield in this combination was significantly higher than in other combinations (*p* < 0.01) (Fig. 2b). Effects of enzymolysis time on protoplast yield were also tested. As digestion time increased from 2 to 6 h, protoplast yield increased, but with further increases in digestion time, protoplast yield decreased significantly (Fig. 2c). These results indicated that the optimal digestion time was 6 h.

Finally, we examined the effect of three tissue types (buds, young leaves and mature leaves) on protoplast yield and found that protoplast yield obtained from young leaves was significantly higher than from the other two types (*p* < 0.01) (Fig. 2d). In summary, the optimal method for protoplast isolation in ‘Yellow River’ is to digest strips of young leaves in a 3% (w/v) Cellulase (R10), 0.8% Macerozyme (R10), 0.04% (w/v) Pectinase, and 0.4 M mannitol enzymolysis solution for 6 h.

### Establishment of a ‘Yellow River’ protoplast transient expression system

**Fig. 3:**
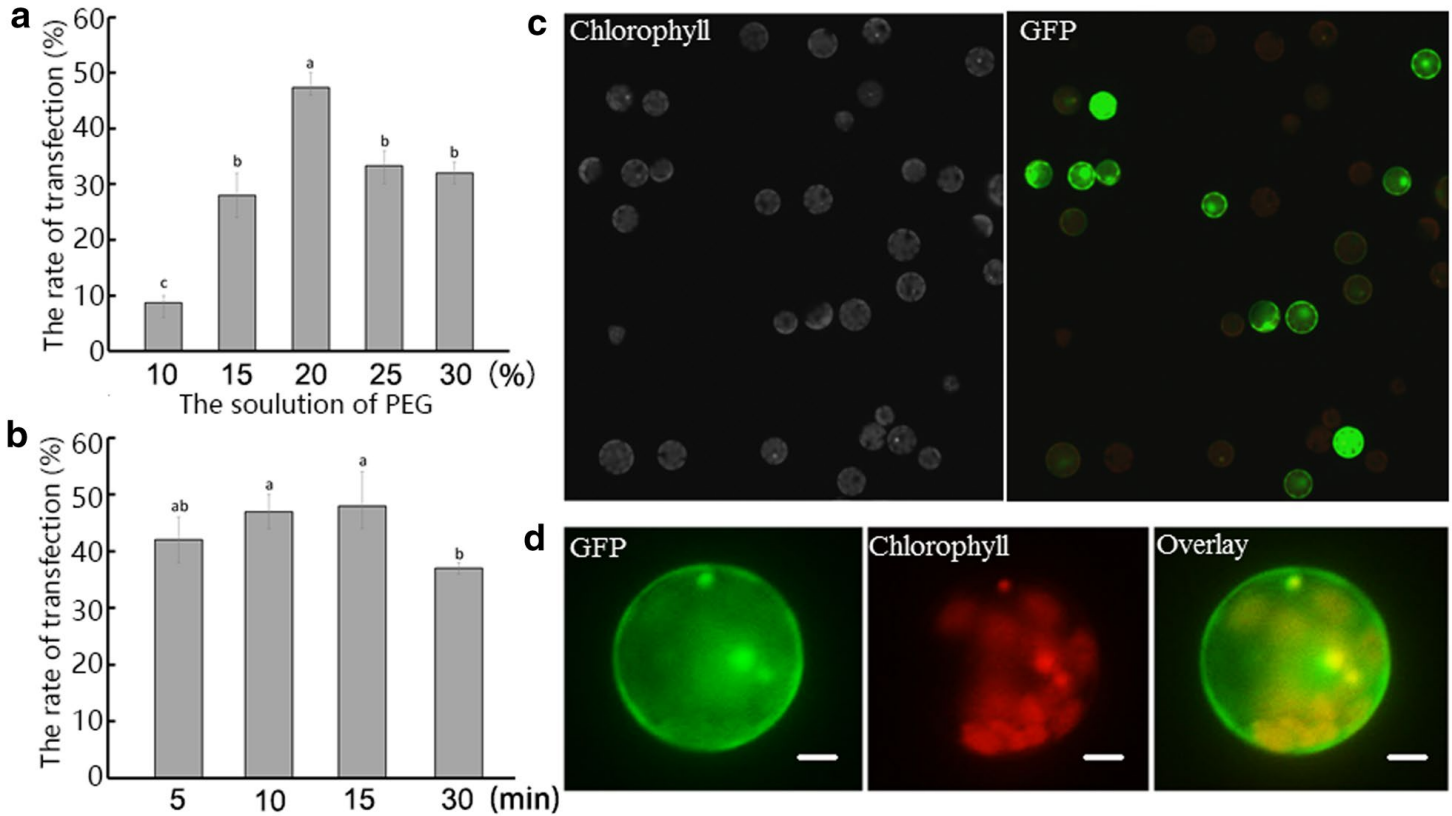
The protoplast transfection ratio and transient expression. The ratio of transfection at 10, 15, 20, 25 and 30% (w/v) PEG4000 concentration (**a**), the ratio of transfection after 5, 10, 15 and 20 min (**b**), images of GFP and chlorophyll autofluorescence (Chl) as well as bright field images of protoplasts captured 12 h after transfection (**c**, **d**). *Bar* in **c**, 50 μm. *Bar* in **d**, 10 μm

We established a PEG-mediated transient expression system for ‘Yellow River’ by testing a range of PEG4000 concentrations and transfection time. The transient expression vector pEZS-NL harboring an EGFP-tag [31] was used to study the transformation efficiency in response to PEG concentrations and transfection time (Fig. 3). The results showed that 20% w/v of PEG4000 was the optimal to get the high transformation efficiency (*p* < 0.01) (Fig. 3a). The transformation efficiency was significantly higher after 10 min and 15 min than after 30 min (*p* < 0.01), and did not differ significantly from efficiency after 5 min (Fig. 3b). Therefore, 5 min was taken as the optimal transfection time.

### Cloning a heat shock transcription factor *MdeHSF1* and its expression pattern under heat stress in ‘Yellow River’

**Fig. 4:**
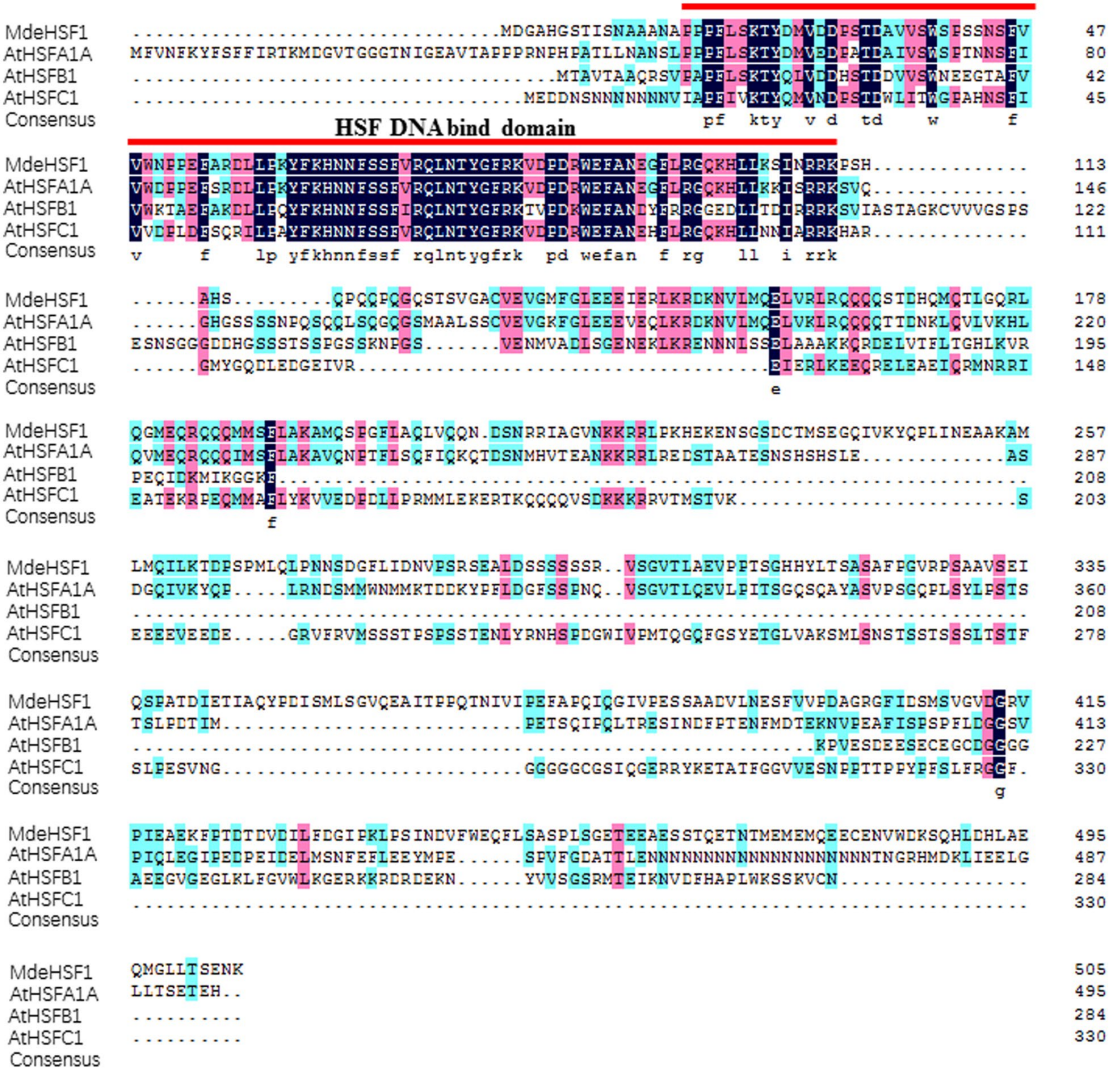
The protein of *MdeHSF1* and three *AtHSF* proteins were compared using DNAman6.0 software. *AtHSFA1A* is *At4g17750*; *AtHSFB1* is *At4g36990*; *AtHSFC1* is *At3g24520*

**Fig. 5:**
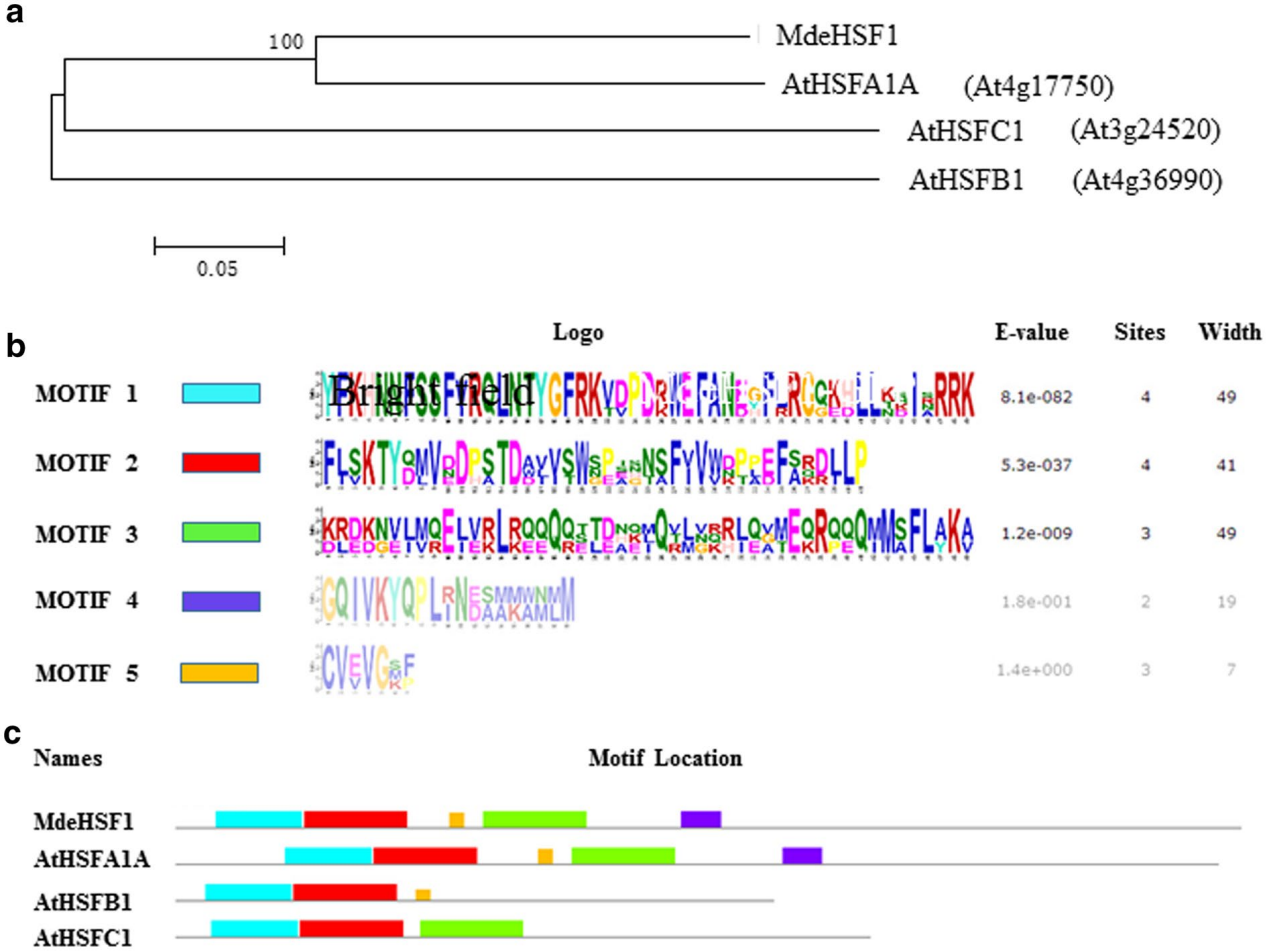
The HSF DNA binding domain is underlined (**a**). Phylogenetic relationship between *MdeHSF1* and *AtHSF* proteins drawn using MEGA6.0 (**b**), the conserved domain analysis of the *MdeHSF1* and *AtHSF* proteins using the MEME website (**c**)

The complete sequence of *MdeHSF1* gene was cloned from ‘Yellow River’. The length of ORF was 1521 bp, coding 506 AA, with PI value of 4.97. The gene contained a conserved high HSF-DNA-binding domain at N terminal, belonging to the heat shock transcription factor family (Fig. 4). The phylogenetic tree showed that *MdeHSF1* had the highest homology to *AtHSFA1A* (Fig. 5a). Furthermore, the subcellular localization of *MdeHSF1* was also predicted to be in the cell nucleus, a typical characteristic of transcription factors (Fig. 5b).

**Fig. 6:**
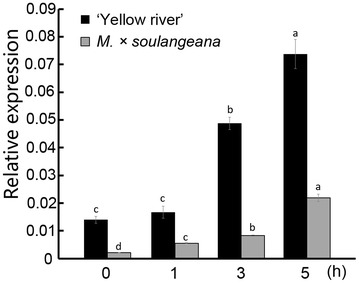
qPCR analysis of the *MdeHSF1* expression level under high temperature (42 °C) from 0 to 5 h in ‘Yellow River’ and *M.* × *soulangeana* leaves

Using expression changes of *MdeHSF1* as a proxy, we estimated the difference in the heat-stress resistance of two species in *Magnolia*, ‘Yellow River’ and *M.* × *soulangeana. MdeHSF1* shared similar expression patterns in two species, exemplified by the increase in expression levels increased with the longer duration of heat stress. The highest expression of *MdeHSF1* in both species occurred after 5 h of heat stress. However, the expression of *MdeHSF1* was more induced by heat stress in ‘Yellow River’ than in *M.* × *soulangeana*. Higher expression of *MdeHSF1* was consistent with the phenomenon that ‘Yellow River’ exhibited higher heat-stress resistance in nature, than that in *M.* × *soulangeana* (Fig. 6).

### Subcellular localization analysis of *MdeHSF1* in ‘Yellow River’ protoplast

**Fig. 7:**
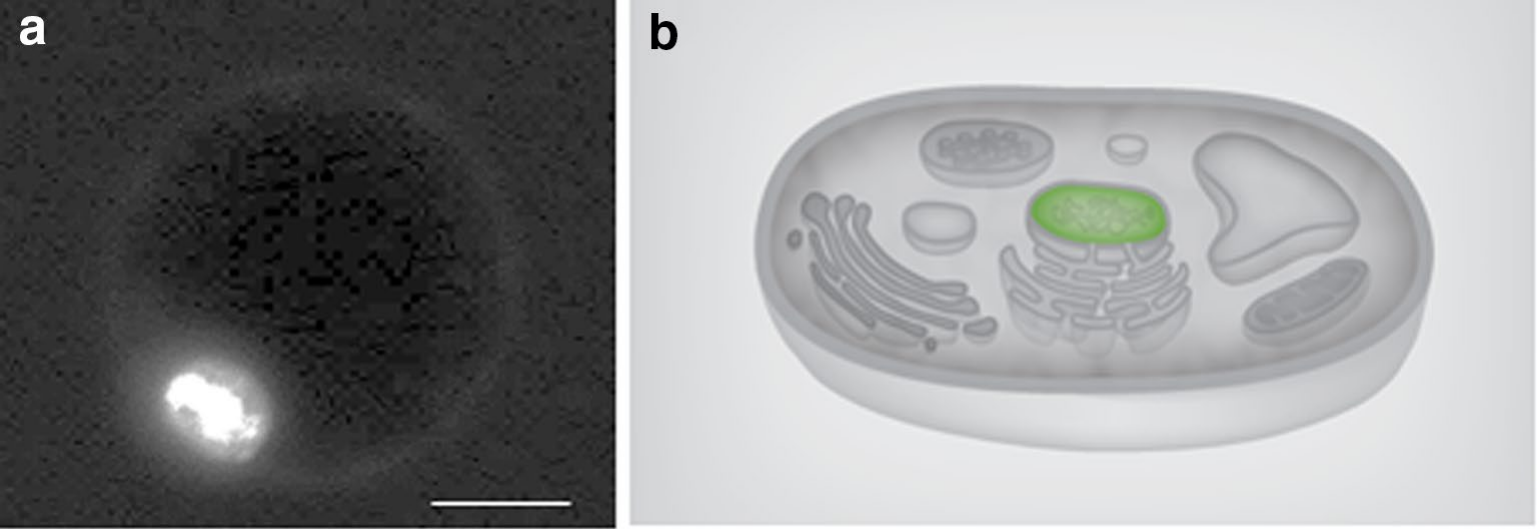
Plasmids can be transferred to protoplasts for transient expression of *MdeHSF1*. The protected picture of the subcellular location of *MdeHSF1* (**a**), the subcellular location of *MdeHSF1* in ‘Yellow River’ protoplast (**b**) *Bar* 10 μm

To determine the subcellular localization of *MdeHSF1*, an *MdeHSF1*-*EGFP* fusion protein vector, *pESZ*-*NL*-*MdeHSF1*, was constructed. Protoplast isolation and transformation was performed as previously described, and the *MdeHSF1* transient expression vector was successfully expressed in the ‘Yellow River’ protoplast (Fig. 7a). These results confirmed that *MdeHSF1* was expressed in the cell nucleus (Fig. 7b), and demonstrated the utility of the protoplast isolation and transient expression system we developed.

## Discussion

Protoplast isolation and transient expression is widely used in plant molecular research to examine gene function, subcellular position and protein–protein interactions. In *Arabidopsis*, the protoplast transient expression system is very mature, and some rapid and simple methods have been developed. For example, the Tape Sandwich system, allows for rapid isolation of high-quality protoplasts from *Arabidopsis* leaves [3]. There are also efficient protoplast isolation and transient expression systems developed for other model plants and economically important plants, such as *Nicotiana tabacum* [33], *Oryza sativa* [16, 34], *Malus* × *domestica* [35] and *Fragaria* × *ananassa* [36]. These protoplast transient expression systems have been used in a variety of experimental studies and have facilitated the advancement of gene function research [16, 34] In contrast, there are a limited number of reports on protoplast isolation and transient expression in ornamental plants. In this study, we developed an efficient protoplast isolation and transient expression system for ‘Yellow River’ after testing different tissue types, enzymatic combinations, enzymolysis solution osmotic potentials, and enzymolysis time. Similar to previous reports on other plants, the effective concentration of PEG solution for the transient expression system is approximately 20%. Under optimal condition, the protoplast yield of ‘Yellow River’ young leaves can reach approximately 1.89 × 10^5^ protoplasts/g FW. This value was higher than the protoplast yield of *Arabidopsis*, *Oryza sativa* and *Populus* [3, 20, 29, 34]. Compared with mature leaves, the young leaves grown in vitro or in a lab, are more suitable to isolate protoplasts.

With continued rise in temperatures globally, plants are increasingly exposed to high temperatures. This is more of a problem for *Magnolia* as it is often used in urban landscaping. Compared with other Magnolia species, such as *M.* × *soulangeana*, ‘Yellow River’ has higher tolerance to heat stress, but the molecular mechanism has remained unclear. Several studies showed that heat-shock transcription factors (HSF) are central regulators of heat-stress responsive genes. The plant HSF family shows a striking multiplicity, showing a strong diversification of expression pattern and function within the family [37]. In plants, there are three classes in the HSF protein family (classes A, B, and C), which are differentiated by peculiarities of their flexible linkers and HR-A/B regions [38]. And many researchers showed that *AtHSFA1A* was overexpressed in the heat shock response [39–41]. *HSFs* trigger the expression of Heat-shock Protein (HSP) in response to various stresses in living organisms, and makes *HSP* complex [40]. In the present paper, one *HSF* transcription factor, *MdeHSF1* was cloned, and we found that the expression level of *MdeHSF1* was much higher in ‘Yellow River’ than that of *M.* × *soulangeana* under high temperature stress. This finding indicates that *MdeHSF1* might be an important gene regulator controlling heat tolerance in ‘Yellow River’. Furthermore, the protein–protein interaction prediction results suggest that *MdeHSF1* exhibits homology with *AtHSFA1A* and plays an important role in the cell signal network.

## Conclusion

These results not only verify the efficiency of the protoplast transient expression system but also provide important clues to understanding heat tolerance in ‘Yellow River’. In future studies, responses of proteins to the expression level of *MdeHSF1* should be explored in more detail and the use of the protoplast system for detecting protein–protein interactions via bimolecular fluorescence complementation assays, could be investigated.

## Additional file

**Additional file 1: Table S1**. Injury degree of landscape plants in the parks and roads in Hang Zhou, China under nature high-temperature. **Table S2**. The sources of all relative reagents
