# Supplementary material for: Efficient isolation of Magnolia protoplasts and the application to subcellular localization of MdeHSF1
Source: Plant Methods. 2017 May 23;13:44. doi: 10.1186/s13007-017-0193-3 (PMC5442663; doi:10.1186/s13007-017-0193-3)
Supplement: Supplementary file 1 — Additional file 1: Table S1. Injury degree of landscape plants in the parks and roads in Hang Zhou, China under nature high-temperature. Table S2. The sources of all relative reagents [file 13007_2017_193_MOESM1_ESM.docx]

**Table S1 Injury degree of landscape plants in the parks and roads in Hang Zhou, China under nature high-temperature**

（From August 26,2013 to September 1,2013 ）

| **Damage Degree** | **Plant Name** | |
| --- | --- | --- |
| **I** | Tree | *Michelia chapensis* |
|  | Bush | [*Viburnum odoratissimum* var. *awabuki*](http://frps.eflora.cn/frps/Viburnum%20odoratissimum%20var.%20awabuki) *Rhododendron simsii, Paeonia suffruticosa, Euonymus japonicus ׳*Ovatus Aureus׳*, Camellia sasanqua* |
|  | Herbage | *Ophiopogon bodinieri, Ophiopogon japonicus* |
| **II** | Tree | ***Magnolia × soulangeana****, Metasequoia glyptostroboides, Taxodium ascendens , Taxodium distichum* |
|  | Bush | *Acer palmatum* ׳Atropurpureum׳*, Aucuba japonica、Fatsia japonica, Ligustrum × vicaryi, Ligustrum quihoui* |
|  | Herbage | *Reineckia carnea* |
| **III** | Tree | ***Magnolia denudate****,* ***Magnolia denudata* ‘Yellow River’** |
|  | Bush | *Sabina chinensis, Sabina procumbens, Acer palmatum* |
|  | Herbage | *Iris tectorum, Paeonia lactiflora* |
| **IV** | Tree | *Platanus acerifolia, Acer buergerianum, Liquidambar formosana, Pinus parviflora, Podocarpus macrophyllus , Pinus massoniana, Osmanthus fragrans, Zelkova serrata, Liriodendron chinense., Magnolia grandiflora, Cerasus yedoensis , Prunus cerasifera* f. *atropurpurea, Armeniaca mume, Malus × micromalus, Malus halliana, Chaenomeles speciose*, *Amygdalus persica* var.*persica* f.*duplex*, *Amygdalus persica*, *Salix babylonica*, *Elaeocarpus glabripetalus* var. *glabripetalus*, *Pistacia chinensis*, *Pterocarya stenoptera*, *Sophora japonica* |
|  | Bush | *Michelia figo*, *Ilex cornuta*, *Hypericum monogynum*, *Hydrangea macrophylla*, *Weigela florida*, *Primula poissonii*, *Jasminum mesnyi*, *Ligustrum japonicum ׳*Howardii׳, *Mahonia bealei , Mahonia fortune*, *Euonymus fortune*, *Camellia japonica*, *Distylium racemosum*, *Photinia serrulata ׳*Red Robin׳, *Rosa multiflora*, *Pyracantha fortuneana*, *Edgeworthia chrysantha*, *Berberis thunbergii*, *Pachysandra terminalis*, *Parthenocissus tricuspidata* |
|  | Herbage | *Musa basjoo*, *Chlorophytum comosum*, *Liriope cymbidiomorpha*, *Oxalis corymbosa, Saxifraga stolonifera*, *Aspidistra elatior* |
| **V** | Tree | *Koelreuteria paniculata*, *Sapindus mukorossi*, *Cedrus deodara*, *Cinnamomum camphora*, *Magnolia grandiflora*, T*rachycarpus fortunei, Celtis julianae, Phoebe sheareri, Ligustrum lucidum, Photinia davidsoniae, Albizia julibrissin, Salix matsudana, Populus tomentosa* |
|  | Bush | *Lagerstroemia indica, Punica granatum, Hibiscus syriacus, Campsis grandiflora, Loropetalum chinense* var*. rubrum, Abelia biflora, Chimonanthus praecox, Wisteria sinensis, Cercis chinensis, Ilex cornuta, IIex chinensis, Nerium indicum, Chaenomeles japonica, Gardenia jasminoides, Forsythia suspense, Buxus sinica, Duranta repens , Trichoglottis rosea* var. *breviracema, Nandina domestica, Cycas revolute, Ficus pumila, Trachelospermum jasminoides, Mucuna sempervirens , Hedera nepalensis* var.*sinensis* |
|  | Herbage | *Portulaca grandiflora, Celosia cristata, Lycoris radiate, Verbena hybrid, Salvia splendens , Canna indica, Petunia hybrid, Setaria palmifolia, Zoysia japonica* |

**Note:**

**I:** Leaves and shoots were damaged more seriously. The Injured area and numbers are more than 50%, some of them were even died.

**II:** Leaves and shoots were damaged worse. The Injured area and numbers are about 30%-50%.

**III**: Leaves and shoots were better than above. The Injured area and numbers are about 15%-30%.

**IV**: Leaves and branches burned, and the injured area and numbers are less than 15%.

**V**: Leaf appeared scorching, the injured area and numbers are less than 5%.

**Table S2 The sources of all relative reagents**

| No. | Name | Company Name |
| --- | --- | --- |
| 1 | cellulose R10 | Yakult Pharmaceutical Ind. Co., Ltd., Japan |
| 2 | HgCl_2_ | Sigma, cat. no. 215465 |
| 3 | macerozyme R10 | Yakult. JAPAN 100mg |
| 4 | pectinase | Yakult Pharmaceutical Ind. Co., Ltd., Japan |
| 5 | mannitol | Sigma, cat. no. M4125 |
| 6 | sorbitol | Sigma, cat. no. M4125 |
| 7 | KCl | Sigma, cat. no. P3911 |
| 8 | MES-KOH (pH 5.7) | MES, Sigma, cat. no. M8250 |
| 9 | CaCl_2_ | Sigma, cat. no. C7902 |
| 10 | β-mercaptoethanol | Sigma, cat. no. M6250 |
| 11 | bovine serum albumin | Sigma, cat. no. A8531 |
| 12 | PEG4000 | Fluka, cat. no. 81240 |
| 13 | Pasmid isolation Kit | Isolated by PureLink® Quick Plasmid Miniprep Kit, Invitrogen, cat. no. K210010 |
| 14 | RQ1 DNase | Promega, Madison, WI, USA |
| 15 | the iScript cDNA Synthesis kit | Bio-Rad, Hercules, CA, USA |
| 16 | DNase I | Invitrogen, CA, USA |
| 17 | the SuperScript reverse transcriptase | Invitrogen, CA, USA |
| 18 | the pMD19-T simple vector | TakaRa, cat. no. 3271 |
| 19 | T4 DNA Ligase | NEB, cat. no. M0202 |
| 20 | PureLink® Quick Plasmid Miniprep Kit | Invitrogen, cat. no. K210010 |
